# Optical coherence tomography angiography in herpetic leucoma

**DOI:** 10.1186/s12880-022-00747-z

**Published:** 2022-02-03

**Authors:** Inês Almeida, Libânia Dias, Jeniffer Jesus, Inês Fonseca, Maria João Matias, João Carlos Pedro

**Affiliations:** 1 Department of Ophthalmology, Centro Hospitalar Entre Douro e Vouga, Rua Dr. Cândido de Pinho, 4520-211 Santa Maria da Feira, Portugal; 2 Department of Orthoptics, School of Health, Polytechnic of Porto, Porto, Portugal

**Keywords:** OCTA, Corneal neovascularization, Herpetic leucoma, Cornea

## Abstract

**Background:**

Herpes simplex virus (HSV) keratitis remains a leading infectious cause of blindness worldwide. Although all forms of HSV keratitis are commonly recurrent, the risk is greatest in stromal keratitis, which is the most likely to result in corneal scarring, thinning, and neovascularization. Recent studies showed the ability of Optical Coherence Tomography Angiography (OCTA) to detect and study vascular abnormalities in the anterior segment, including abnormal corneal vessels. This study intends to investigate the potential of OCTA device to image and describe quantitatively the vascularization in eyes diagnosed with herpetic leucoma and to discuss and review the usefulness of this technique in this pathology.

**Methods:**

A Cross-sectional study was made, including 17 eyes of 15 patients with leucoma secondary to herpetic keratitis. All eyes underwent anterior segment Slit-Lamp photography (SLP), and OCTA with en-face, b-scans and c-scans imaging. The vessel density (VD) was analyzed in the inferior, nasal and temporal corneal margin in all patients, and in the central area, in eyes with central corneal neovascularization (CoNV). The measurements were calculated after binarization with ImageJ software, using OCTA scans with 6 × 6 mm in a depth of 800 μm.

**Results:**

Patients included had a mean age 53.267 ± 21.542 (years ± SD). The mean total vessel area was 50.907% ± 3.435%. VD was higher in the nasal quadrant (51.156% ± 4.276%) but there were no significant differences between the three analyzed areas (p = 0.940). OCTA was able to identify abnormal vessels when SLP apparently showed no abnormal vessels; OCTA was able to distinguish between larger and smaller vessels even in central cornea; OCTA scans allowed the investigation of several corneal planes and the relation of them with clinical findings.

**Conclusions:**

OCTA can be useful in both qualitative and quantitative follow-up of patients and may become a non-invasive alternative to objectively monitor treatment response in eyes with corneal vascularization due to herpetic infection.

## Background

Herpes simplex virus (HSV) is endemic in virtually every human society throughout the world. Humans are the only natural reservoirs for the virus that spread from sites of initial infection to establish latent infection, forming a unique long-term relationship with their host [1, 2]. In fact, HSV keratitis remains a leading infectious cause of blindness worldwide mostly because of its recurrent nature [3]. Recent publications have shown that, in industrialized societies, the progressively delayed acquisition of HSV-1 may lead to an increase in the occurrence of primary HSV-1 infection as an adult, rather than as a child [1].

Herpetic ocular disease may be classified as primary or recurrent and may also be classified as blepharitis, conjunctivitis, epithelial keratitis, stromal keratitis, iridocyclitis, or retinitis based on the inflamed tissue [2]. Since antivirals cannot eliminate or block the establishment of latent HSV infection, it is the relapsing and recurring stromal and endothelial disease that renders the greatest morbidity through corneal scarring and neovascularization [3, 4].

In fact, corneal opacification arise from the complex interplay between viral activity and the host immune response and the subsequent inappropriate blood vessel formation in the normally transparent and avascular cornea [4]. As it is well known corneal neovascularization (CoNV) is a pathological condition which compromises the immune privilege state of the cornea, reduces corneal transparency, causes vison loss and subsequently increases the rate of graft rejection after corneal transplantation [5].

Since its first in vivo use for the retina, optical coherence tomography (OCT) imaging has revolutionized the ability to evaluate the eye and its structures with unprecedent resolution [6, 7]. Recent technological developments increased sensitivity and speed of OCT systems, and so it is now possible to delineate blood vessels using OCT by using decorrelation between consecutive scans. The basis of OCT angiography (OCTA) is essentially comparing consecutive B-scans, which is now possible with B-scan rates of several hundred hertz (Hz)—optimal for detecting flow in the microvasculature of the eye [6, 7].

Currently assessment of the corneal and anterior segment (AS) vasculature is constrained to slit lamp photography (SLP) or angiography techniques. Image analysis software to analyze photographic images has been described but the analysis is time-consuming and color photographs have limited visualization of vessels in the presence of corneal edema, deposits or scar, even more so for very small caliber or deeper layer vessels [5, 6]. Fluorescein and indocyanine green angiography techniques have demonstrated better vessel delineation than SLP despite the presence of corneal scars. However, both techniques are invasive and expose subjects to common gastrointestinal side effect or serious adverse reaction like anaphylactic shock [5]. Originally designed for the retina, OCTA imaging system was adapted for normal AS vasculature and has been described as a potential rapid, non-invasive technique to diagnosis and monitoring the treatment of scleral and corneal diseases [8, 9]. Therefore, this study was conducted to explore the potential of OCTA device to image and describe quantitatively corneoscleral margin vascularization in eyes diagnosis with herpetic leucoma and to discuss and review the usefulness of this technique in this pathology.

## Materials and methods

Patients with herpetic leucoma diagnosed by a Corneal Expert at Centro Hospitalar Entre Douro e Vouga, Santa Maria da Feira, Portugal were prospectively recruited for a cross-sectional study of vessel density (VD) measurements with OCTA. The study has been performed in accordance with the Declaration of Helsinki and its later amendments, and ethics approval was obtained from the internal Ethical Committee. All patients completed and signed informed consent forms.

The study included 17 eyes of 15 patients with leucoma secondary to herpetic keratitis for more than 3 years and without crises for at least 6 months. Exclusion criteria included the presence of congenital eye disorders, other causes of corneal opacity and low-quality images obtained with OCTA.

Demographics and clinical data (age, sex, involved eye, best-corrected visual acuity [BCVA], tonometry, slit-lamp biomicroscopy of the anterior and posterior segment of the eye) were evaluated. After that all patients underwent imaging. First an AS SLP was taken using digital slit-lamp camera system (SL-D Digital Slit-Lamp; Topcon, Tokyo, Japan) with a standard diffuse illumination (× 10 magnification, 45° angle) and then OCTA images of the inferior, nasal and temporal corneoscleral margin were obtained. VD measurements were obtained by binary vessel maps generated from OCTA slabs analyzed using ImageJ software.

### OCT angiography

OCT angiograms of the AS were taken by the spectral domain OCTA system Avanti XR AngioVue (Optovue, Inc., Fremont, CA, USA) using the Angio Retina mode (split-spectrum amplitude-decorrelation angiography), adapted for AS imaging by coupling the long cornea adaptive module lens (CAM-L; Optovue, Inc).

**Fig. 1:**
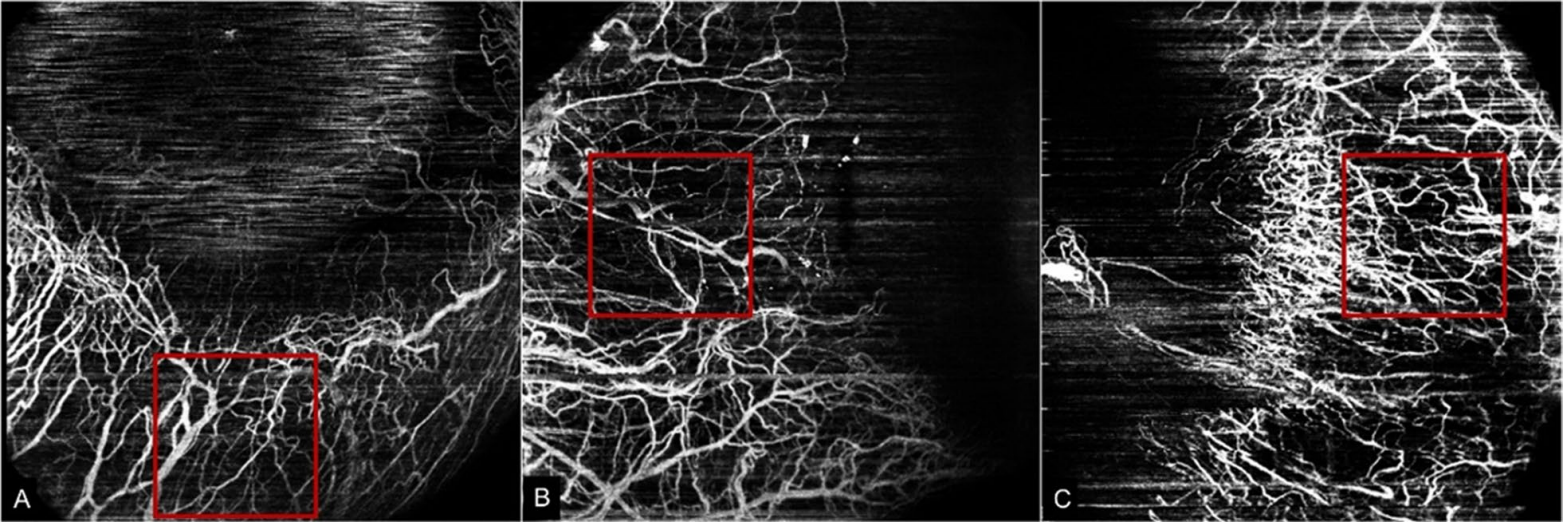
Representative region of interest (ROI) in the OCTA slabs. The red line square shows the 2.5 × 2.5 mm (150 × 150 pixels) ROI used to assess the vascular density of corneoscleral margin in the inferior quadrant (**A**), temporal quadrant (**B**) and nasal quadrant (**C**). In this case, right eye was analyzed

Blood flow is detected through the decorrelation signal generated from the red blood cell motion through consecutive A-scans of the same location, generating a volumetric blood flow analysis. All OCTA scans were conducted by one expert professional operator and performed as described in previous studies [6, 9, 10]. After correct positioning of the patient at the device and turning down the background illumination, a manual adjustment was done for clear imaging the AS, moving the cornea adaptor lens until the corneal surface appeared in the OCT window (approximately 2 to 3 cm). The subjects were instructed to look in different positions to expose the different parts of the conjunctiva: a fixation point was placed on the right, left and on top of the patient. The patient fixated these points when the image was acquired—to fully expose the inferior conjunctiva, the subjects gazed upon the fixation point located superiorly; to fully expose nasal conjunctiva, the subjects looked at the fixation point located on the temporal side of the eye; to fully exposed temporal conjunctiva, the subjects were invited to fixate the point located on the nasal part of the eye. Due to the lack of a standard protocol for imaging of the AS OCTA, 6 × 6 mm scans (250 × 250 pixels) to a depth of 800 μm were performed in inferior, nasal and temporal quadrants along the limbus. A square of 2.5 × 2.5 mm (150 × 150 pixels) tangent to the corneoscleral margin, which comprises the most part of corneoscleral margin vascular networks, at 3 o’clock position in the right eye and 9 o’clock position in the left eye, and at 6 o’clock bilaterally was defined as the representative region of interest (ROI) (Fig. 1). The ROI for VD measurement were validated by two different researchers.

### Images processing and vessel density measurements

**Fig. 2:**
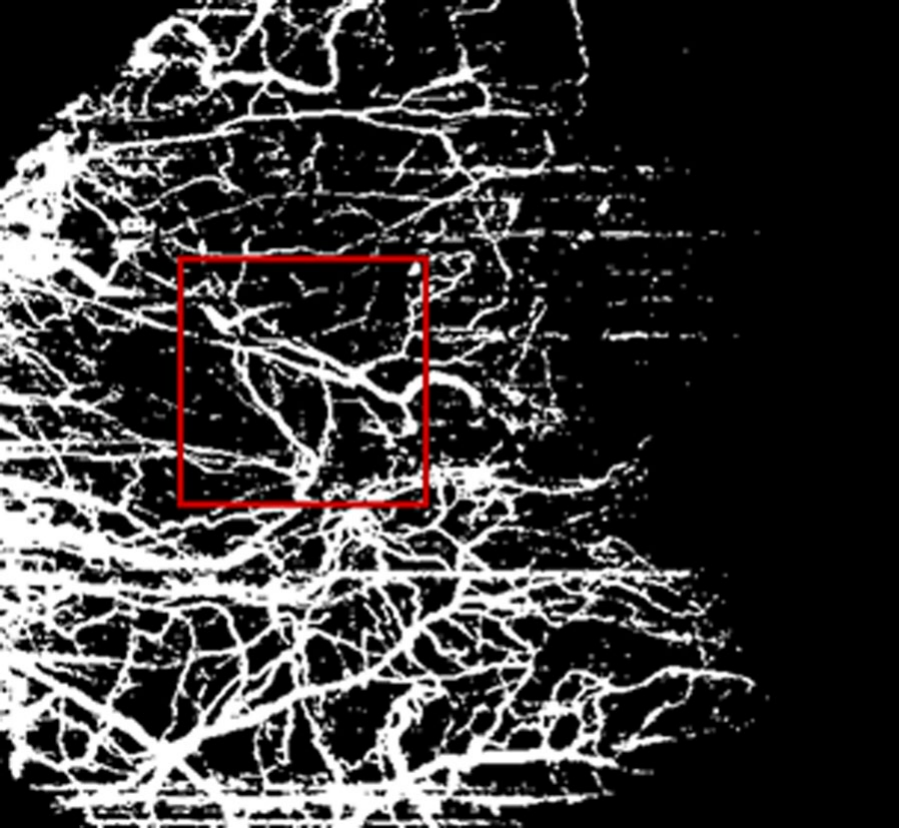
Illustration of binary image generated by ImageJ software for analysis. Vessel pixels = white pixels; background = black pixels. Red square indicating the ROI

VD was defined as the percentage area occupied by the large vessels and microvasculature in the analyzed region. The OCTA images were exported from the equipment in Portable Network Graphics (PNG) image file for analysis, into the Fiji Software, a distribution of the open-source Java-written software *ImageJ*, (V.1.49p, National Institutes of Health, Bethesda, Maryland, USA) [11]. To calculate the VD, the OCTA images were binarized and were converted to an 8-bit image for processing (Fig. 2). The program adjusts threshold with *Shanbhag* method applied to improve quality image, by highlighting blood vessels and reducing the noise. The contrast of the images was adjusted using the Equalize Histogram of this algorithm. VD was expressed in percentage by taking the ratio of the total vessel area to the total area of the analyzed region (area with the highest number of whites—vessel pixels = 1; background = 0). Only clear images were studied, and the images of poor quality (image quality index < 3) caused by motion artifacts (poor fixation and blinking artifacts) were excluded.

### Statistical analysis

Statistical analysis was performed with the Statistical Package for Social Sciences (SPSS for Mac, version 25; IBM/SPSS, Chicago, IL). The main outcome parameters included the percentage of corneoscleral margin VD detected by OCTA in different quadrants (inferior, nasal and temporal), expressed as mean ± SD. The normality of the sample distribution was confirmed using the Shapiro–Wilk test (p > 0.05). The homogeneity of variances was calculated by *Levene* Test. The One-Way ANOVA test was used, with Post-hoc *Bonferroni Test*, to analyze the measurements in each quadrant. Correlation analysis between the values in the different locations were investigated by *Pearson’s* correlation test. *p value* ≤ 0.05 was considered of statistical significance.

## Results

The study included a total of 17 eyes (10 right eye, 7 left eye) of 15 patients (3 females, 12 males) with mean age 53.267 ± 21.542 (years ± SD). The demographic and clinical characteristics of the participants are summarized in Table 1. All had history of herpetic leucoma for more than 3 years and had no crisis for at least 6 months; Some patients had other conditions with visual impact: 4 patients with cataract, 4 patients with phaco surgery history and 1 patient with bilateral amblyopia. It was made an OCTA image capture of the inferior, nasal and temporal vessels of corneoscleral margin within each ROI. There was one eye where the OCTA didn´t capture the nasal and temporal areas due to poor gaze fixation and resultant poor quality images.

**Table 1:** Demographic and clinical parameters of the study subjects

	Age (years)	BCVA (logMAR)	IOP (mmHg)
n (valid)	15	17	17
Mean ± SD	53.267 ± 21.542	0.794 ± 0.952	15.5 ± 2.382
Minimum	13	+ 3.0	12
Maximum	81	0.0	20

*BCVA* best-corrected visual acuity, *IOP* intraocular pressure

Statistical analysis are present in Tables 2, 3 and 4. The mean total VD in all quadrants analyzed was 50.907% ± 3.435% (Table 2). The mean VD was higher in the nasal quadrant (51.156% ± 4.276%) and lower in the temporal quadrant (50.74%1 ± 3.017%), but there were no significant differences among the three areas (p > 0.05), when multiple comparisons were performed (Tables 2 and 3). There was a positive correlation between nasal and temporal mean VD (r = 0.490, p < 0.05) (Table 4).

**Table 2:** Descriptive statistics of the groups and normality check of the variables

Area	Mean VD (%)	SD	Minimum	Maximum	Shapiro–Wilk	p-value of Shapiro Wilk
Inferior	50.829	3.104	46.380	58.461	0.928	0.138
Nasal	51.156	4.276	43.954	59.905	0.970	0.758
Temporal	50.741	3.017	42.326	55.425	0.906	0.053
Total	50.907	3.435	42.326	59.905	-	-

*VD* vessel density, *SD* standard deviation

**Table 3:** Multiple comparisons between all the quadrants analyzed—One-Way ANOVA test with Post-hoc *Bonferroni Test*

Bonferroni	Quadrant	Mean difference (I–J)	Std. error	Sig	95% confidence interval for difference^a^	
					Lower bound	Upper bound
Between groups		–	–	.801	–	–
Inferior^a^	Nasal	− .034	.808	1.000	− 2.026	1.959
	Temporal	− .483	.808	1.000	− 2.476	1.510
Nasal^a^	Inferior	.034	.808	1.000	− 1.959	2.026
	Temporal	− .449	.808	1.000	− 2.442	1.543
Temporal^a^	Inferior	.483	.808	1.000	− 1.510	2.476
	Nasal	.449	.808	1.000	− 1.543	2.442

Based on estimated marginal means

^a^Adjustment for multiple comparisons: Bonferroni

**Table 4:** Correlations between the variables

	Temporal	Inferior	Nasal
Pearson’s r	–		
Temporal	–		
p-value			
Pearson’s r	0.411	–	
Inferior	0.114	–	
p-value			
Pearson’s r	0.49	0.374	–
Nasal	0.054*	0.154	–
p-value			

*Statistical significant (p < 0.05)

**Fig. 3:**
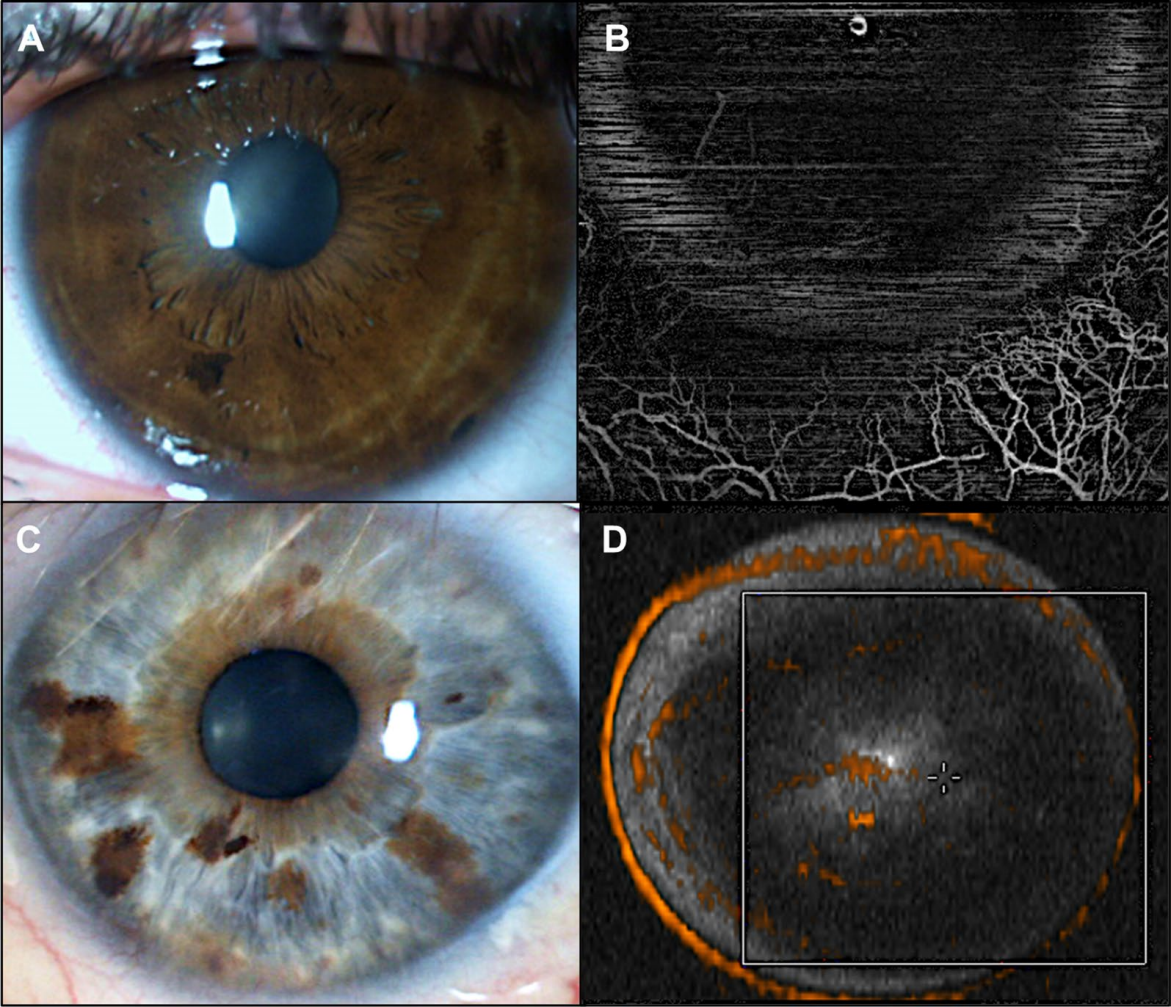
Herpetic leucoma visualized in AS SLP without visible corneal vessels (**A**) with abnormal corneal and limbal vasculature distinguished in the corresponding OCTA image (red arrows) (**B**). Light dense leucoma without visible corneal vessels (**C**) in AS SLP; OCTA en face image can detect flow within vessels indistinguished in the AS SLP (**D**)

**Fig. 4:**
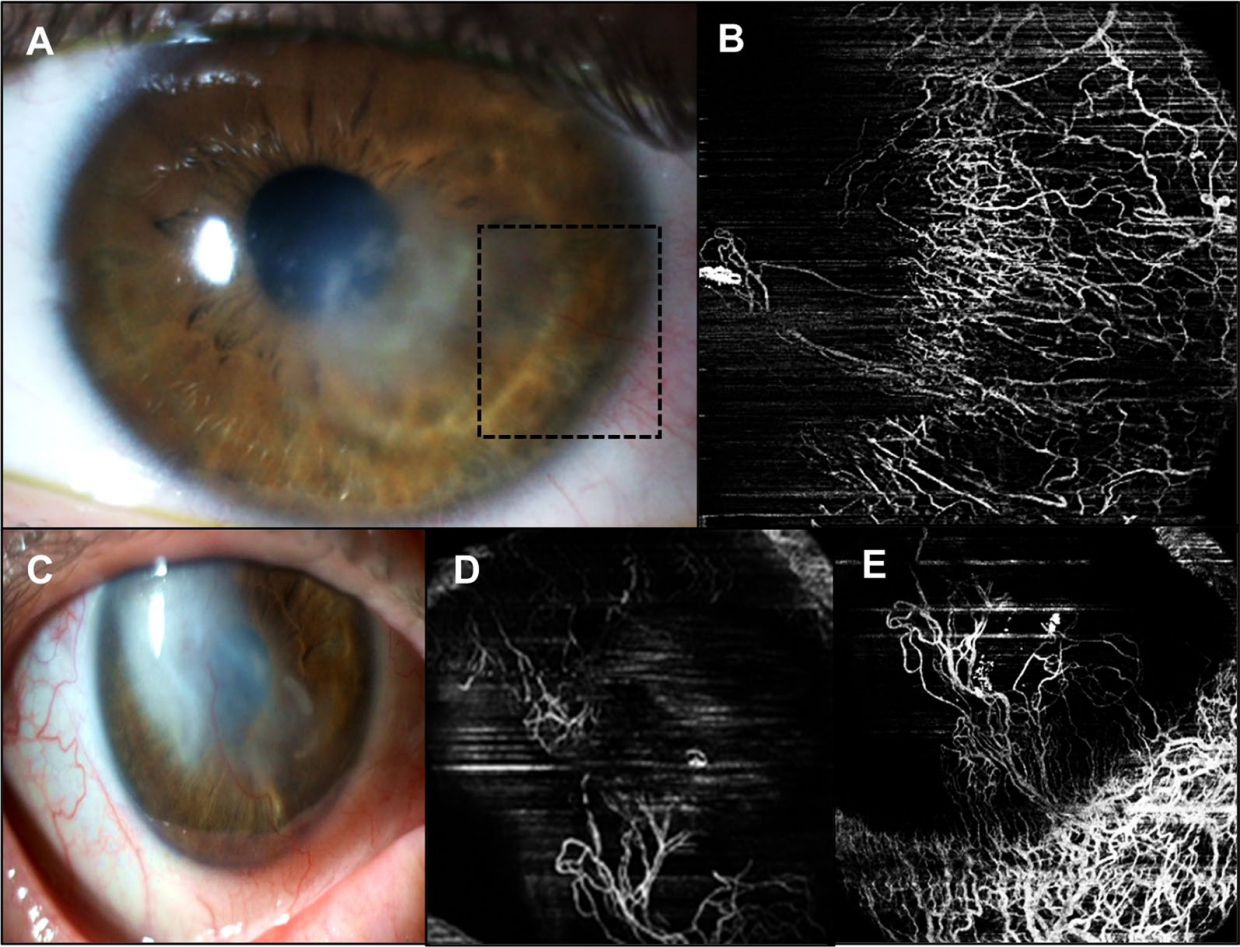
Example of CoNV invading corneal stroma in AS SLP (**A**, **C**). Abnormal vascularization is clearly image by OCTA (**B**, **D**, **E**) as well as other imperceptible corneal vessels and abnormal corneal vascular arcade. Scans obtained at most superficial level

All eyes underwent an AS SLP. These photographs were compared qualitatively with OCTA images to demonstrate some advantages of this technique. Figure 3 illustrates two cases where OCTA was able to identify abnormal vessels. The first case (Fig. 3 A, B) corresponds to the older patient of this study with a temporal paracentral herpetic leucoma of the right eye. In the AS SLP there are apparently no abnormal vessels (A); OCTA image localize vessels in the temporal quadrant (B). The second case (Fig. 3 C, D) corresponds to a 64-year-old male without apparent abnormal corneal vessels (C); OCTA scans were able to detect flow within vessels in the central cornea (D). Figure 4 exemplifies the cases of two patients with bilateral disease with CoNV invading corneal stroma in AS SLP (Fig. 4A, C). OCTA has the ability to demonstrate the extent of perceptible and imperceptible CoNV invading corneal stroma neovascularization, discriminating between larger and smaller vessels as well as abnormal corneal vascular arcade (Fig. 4B, D, E).

**Fig. 5:**
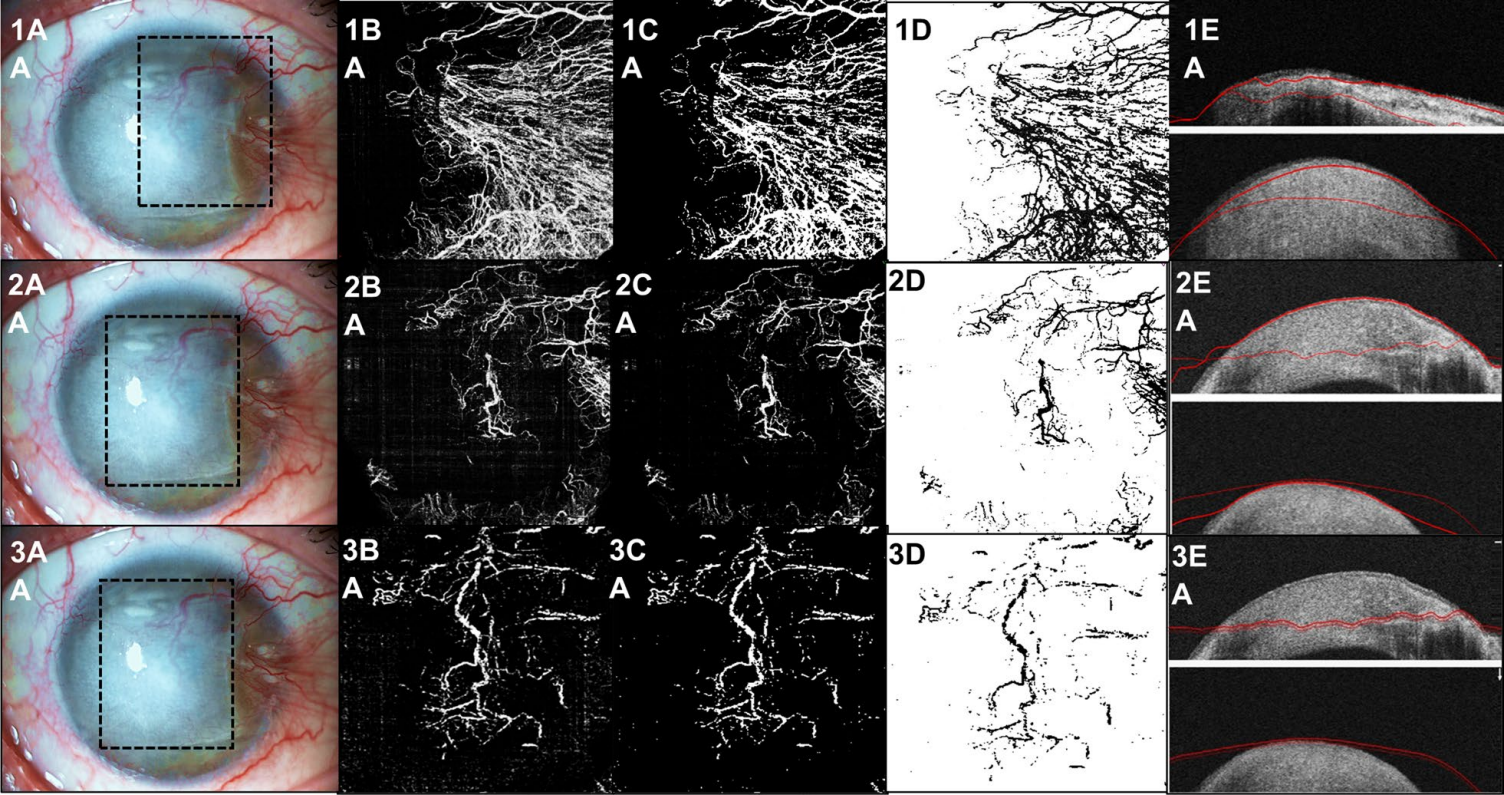
Example of a dense cornea herpetic leucoma with abnormal neovascularization visualized in AS SLP (**1A**, **2A**, **3A**). OCTA image is able to demonstrate the extent of neovascularization at nasal side and to discriminate between larger and smaller vessels (**1B**). Images are imported as 8-bit color images in the ImageJ software, and the contrast is enhanced to reduce the surrounding noise (**1C**). The image was converted to binary using locally calculated thresholds (**1D**). Representative localization at the level in which OCTA was acquired (150–200 µm). The C-scans were determined by two parallel lines automatically defined, with the default thickness of 50 µm, with the posterior possibility of manual modifications (**1E**). The same analyze was made to central and anterior neovascularization (50–150 µm) (**2B**, **2C**, **2D**, **2E**) and central and deep neovascularization (250–300 µm) (**3B**, **3C**, **3D**, **3E**), that seems to have more vessels than anterior evaluation

A study in several corneal planes is also possible. Figure 5 demonstrates a case of a patient 55-year-old with a dense herpetic leucoma and CoNV (1A, 2A, 3A). With en face OCTA function, the C-scans are determined by two parallel lines automatically defined, with the default thickness of 50 µm, with the posterior possibility of manual modifications. This gives the possibility to know the extent of corneal pathology and abnormal vasculature. The analyzes was made to central and anterior neovascularization (50–150 µm) (2B, 2C, 2D, 2E), nasal neovascularization (150–200 µm) (1B, 1C, 1D, 1E) and central and deep neovascularization (250–300 µm) (3B, 3C, 3D, 3E).

## Discussion

The healthy cornea is avascular due to corneal “immune privilege”, a process of homeostasis between the low level of angiogenic and high level of antiangiogenic factors [12]. Inappropriate blood vessel formation in the normally transparent and avascular cornea is a major cause of vision loss and blindness due to herpetic keratitis [1, 4]. Currently, there is no epidemiological study that provides an accurate estimate of the incidence and prevalence of CoNV in the general population [3, 12].

Although mild CoNV may be asymptomatic, more severe forms predispose the cornea to inflammation, lipid exudation and scarring, leading to significant loss in visual function [8]. Clinically, CoNV is subdivided into 3 groups based on the pattern of angiogenic invasion: (1) superficial vascularization, where vessels sprout from the superficial marginal arcade and extend beneath the epithelium; this is commonly seen in stromal keratitis; (2) vascular pannus which results from extension of vessels and fibrous tissues from the limbus onto the peripheral cornea and is mainly seen in ocular surface disorders when an insult is sustained for a long period of time; (3) deep stromal vascularization can occur at any level of the stroma, from beneath Bowman layer to Descemet membrane, as seen in herpetic and luetic interstitial keratitis [12, 13]. Although no one factor can explain all causes of CoNV, 3 pathological mechanisms have been identified: hypoxia, inflammation, and limbal barrier dysfunction. In human HSV type 1 corneal infection recent evidence suggests alteration of the normal balance between angiogenic and anti-angiogenic responses as the likely cause of corneal vascularization [4, 12, 14]. After the first response of production of proinflammatory cytokines and chemokines and an invasion of the cornea by PMN, HSV infection can induce the production of many angiogenic factors such as thrombospondins 1 and 2, vascular endothelial growth factor, matrix metalloproteinases (MMP) 2 and 9, platelet-derived growth factor (PDGF) and beta fibrosing growth factor (bFGF) [4, 12, 15, 16].

Knowing all these described mechanisms, in daily practice, and despite the importance of having an objective way of assessing abnormal corneal vessels, there has not yet a good noninvasive imaging technique to be widely used. Clinical applications are vast and include preoperative localization of CoNV for target intervention, monitoring treatment response using vascularization area or the diagnosis and prognostication of corneoscleral inflammmation [9]. So an essential requirement for disease monitoring and evaluating the efficacy of any potential treatment is the ability to quantify CoNV before and after intervention [8, 17].

SLP of CoNV is limited by inconsistent vessel delineation from frequently coexisting corneal opacification, poor standardization, and the inability to perform quantitative measurements [5]. Angiography techniques utilize intravenous injections of fluorescein and indocyanine green and require long acquisition times, with risks of serious adverse reactions [8]. Since the recent advancement of the technology and its availability for clinical implementation, the interest of OCTA to assess AS vessels has grown rapidly [5]. The group of Ang et al. was a pioneer in presenting the application of OCTA in various clinical conditions with abnormal corneolimbal neovascularization such as postherpetic keratitis scar [6]. Subsequent studies have been published: one have shown the ability to detect small and deep vessels in cases of previous herpetic keratitis [9], other presented a small case series of herpetic corneal vascularization supporting the use of OCTA imaging technique for monitoring vascular changes after a variety of treatments [8], and other showed good agreement and comparable results between OCTA and indocyanine green angiography for measurement of corneal vascularization [18]. Rodriguez-Garcia et al. published the first study analyzing corneal alterations (scarring extension and leukoma depth) observed by spectral-domain -OCT in HSV keratitis: 51.7% of the lesions were located in the visual axis, and 48.3% were located in the paracentral and peripheral cornea; 44.8% of the lesions extended to the mid-stromal depth and had a medium density; and most of the inactive lesions (72.4%) covered an area between 25 and 45% of the ocular surface. As previously mentioned, the inflammatory state behind corneal opacity triggers angiogenesis, with the appearance of new formed blood vessels from the limbal vessels [19, 20]. In this preliminary study, a group of eyes with the same condition was selected to obtain measurements of the corneoscleral vessels using OCTA technology. 17 eyes with residual herpetic stromal scar with/without CoNV were included. It was possible to get a clear visualization of abnormal vessels invading the corneal stroma on the OCTA scans that were not as clearly seen on SLP, also taken in the same day. Farther that OCTA has proven to be an effective tool in quantification of vessels in patients with herpetic leucoma, with a mean total vessel area of 50.907% ± 3.435%. Mean VD was higher in the nasal quadrant (51.156 ± 4.276) but there was no significant differences between the three analyzed areas (p > 0.05). Despite, it was found a positive correlation between the VD in the nasal and temporal quadrants, which means that the more vascularized the nasal quadrante (which was the quadrant with the largest VD), the more vascularized the temporal quadrant tends to be. Additionally, OCTA can also provide simultaneous assessment of depth of the lesion, as well as its associated abnormal blood vessels and abnormal vascular loops. In many herpetic cases deep stromal vascularization occurs as it was mentioned before in this discussion. The en face scans can be in the future a useful instrument for corneal planes study, as it is now for retinal diseases. This article presents an example of this modality using the definitions created for retinal analysis (Fig. 5). This quantitative and qualitative findings can suggest that HSV corneal lesions tend to be extensive, as cited by Rodriguez-Garcia et al., and associated with new vessels; OCTA tends to be a promising method in identifying vasculature anomalies; however more studies are needed. As it is known that HSV CoNV may be prominent, even reaching the central cornea, OCTA may become useful in monitoring patients with acute herpetic crisis and at risk of developing those CoNV.

OCTA technology applied to AS presents some limitations that must be noted. Image distortions may occur due to patient movement, it doesn´t carry an eye-tracking system with registration which is necessary for comparisons in follow-up scans and it is unable to detect vessels without red cell flow. Some scans may contain artifacts that may appear as abnormal vessels, the ability to segment the images to separate the conjunctival vessels from scleral vessels is not optimal and image resolution is also not sufficient to distinguish normal from abnormal vessels. Despite all efforts to avoid bias, some limitations must be noted in this study. Firstly, in the study design, first priority was to understand if there were some alterations in the vascular pattern of patients with herpetic leucoma, being a descriptive pilot study that did not include a comparison group of healthy individuals. Second, although the improvements in the acquisition software of OCTA, the ability to segment and analyze the images was not optimal, being impossible to eliminate some motion artifacts. The lack of a fixation target or eye tracking system led to multiple motion artefacts, so images of poor quality were excluded. Moreover, imaging the superior quadrants of the corneal limbus was difficult due to the lashes, patient collaboration for this localization and bigger time consumption for image acquisition, being necessary an operator to retract the upper eyelid to avoid optical shadows cast by the eyelashes in the scans. So superior corneoscleral margin was not analyzed in this study. Thus, improvements in the segmentation software and regarding motion corrections are needed to enhance the reliability of the data. Besides, although the ROI was validated by two different individuals, the initial annotation of the most vascularized region in each quadrant were made manually. In fact, image analysis needs to improve with time as well, with such semi- automated methods to quantify corneal vascularization and with digital images using threshold analyses and filters (for example the acquisition quality of the C-scans was poor in most eyes). Therefore, many studies, including this one, still rely on subjective scores for corneal vascularization and grading. Finally the authors also recognize the limitations of this pilot study in a small number of eyes to be evaluated with OCTA technique to describe quantitatively the vascularization in eyes diagnosed with herpetic leucoma.

Nonetheless, this research expand the importance of OCTA in the management of patients with this condition. At present these patients are largely monitored with serial photographs, the only viable tool for rapid and non-invasive clinical use; developing standardized OCTA image protocols will help in both qualitative and quantitative patients follow-up and may become a non-invasive alternative to objectively monitor treatment response in eyes with CoNV [7, 8, 21].

## Conclusions

In conclusion, the study presents results that confirm OCTA has an useful technology and highlights its clinical applications in patients with herpetic leucoma, being a stimulus for the development of this new technique to be used routinely in clinical practice, similar to what already happens in the retinal diseases.

## Data Availability

The datasets generated during and analyzed during the current study are not publicly available due to upholding data protection guidelines, but are available from the corresponding author on reasonable request.

## Abbreviations

HSV: Herpes simplex virus
OCTA: Optical coherence tomography angiography
VD: Vessel density
CoNV: Corneal neovascularization
OCT: Optical coherence tomography
AS: Anterior segment
SLP: Slit lamp photography
BCVA: Best-corrected visual acuity
ROI: Representative region of interest
